# Ambient temperature modulates body weight changes in patients with advanced oncological diseases and anorexia cachexia syndrome

**DOI:** 10.1007/s00484-023-02513-4

**Published:** 2023-07-04

**Authors:** Paloma Encinas, Jose Luis Rodriguez-Arias, Luis Miguel Luengo Pérez, Daniel Cortizo, Emilio Gutierrez

**Affiliations:** 1 Departamento de Psicología y Antropología. Facultad de Enfermería y Terapia Ocupacional, Universidad de Extremadura, Cáceres, Spain; 2 Servicio de Salud Mental. Complexo Hospitalario Universitario de A Coruña (C.H.U.A.C.), Coruña, Spain; 3 Departamento de Ciencias Biomédicas, Facultad de Medicina y Ciencias de la Salud, Universidad de Extremadura, Badajoz, Spain; 4 Unidad Venres Clínicos, Servicio de Psicología, Facultad de Psicología, Universidad de Santiago de Compostela, Santiago, Spain; 5 Departamento de Psicología Clínica y Psicobiología, Facultad de Psicología, Universidad de Santiago de Compostela, Campus Vida, 15706 Santiago de Compostela, Spain

## Abstract

**Objective:**

To assess the impact of ambient temperature (AT) on the evolution of bodyweight in patients with heterogeneous types of cancer in advanced stages of the disease (stages III and IV) and anorexia- cachexia syndrome (ACS).

**Methods:**

A prospective naturalistic multicenter study of patients undergoing oncological treatment at four hospitals during a three-year period (2017–2020) in the Autonomous Community of Extremadura in southwestern Spain with a continentalized Mediterranean climate of mild and relatively rainy winters, and particularly hot and sunny summers. Bodyweight changes were obtained from the medical records of 84 oncological patients (59 men and 25 women, age range 37–91 yrs). Mean monthly AT was used to examine the association of weight changes across cold and warm bimesters -BIMs (December and January, vs. July and August), Trimesters -TRIMs (July to September vs. December to February), and Semesters -SEMs (May to October vs. November to April). Weight changes between two consecutive weight measures were categorized as weight gain, weight loss, or no weight change. Differences across cold and warm seasons were analysed using parametric (ANOVA), and nonparametric statistics (Chi-square and binomial z tests). An alpha-rate of 0.05 was used for all analyses.

**Results:**

A weight loss trend was observed during BIMs cold periods in comparison to warm ones (*p* 0.04). However, differences in average bodyweight were not significant. The negative impact of cold periods was more marked in men than in women, (*p* = 0.05; *p* = 0.03, for cold vs. warm BIMs and TRIMs, respectively). In contrast, significantly higher weight gain percentages were found in women during warm TRIMs and SEMs (*p* = 0.03, and *p* = 0.01, respectively). As for the number of patients dying during the study (*N* = 56; 39 men, 17 women), there were a significant interaction between temperature (cold/warm), and mean weight *F (*1, 499) = 6.06, *p* = 0.01, which revealed a pattern of weight loss in the cold semester as opposed to weight gain during the warm SEM months.

**Conclusions:**

AT temperature modulated body weight changes in patients with advanced oncological disease and ACS. Two main limitations of the study were the absence of information on diets as a moderating factor of weight loss/gain, and the lack of the patients’ weight measurements closest to the date of diagnosis prior to admittance to the study. As for the practical implications, it remains to be seen whether an adjunctive heat supply will serve a buffering effect on weight loss during colder seasons for patients with advanced cancer and ACS.

**Supplementary Information:**

The online version contains supplementary material available at 10.1007/s00484-023-02513-4.

Cancer patients exhibit an array of symptoms owing to the disease itself and the treatments administered. Among them, weight loss is present from the onset of the disease and has both prognostic and predictive relevance for oncologic patients (Mariani et al. 2012). For oncological patients, a good nutritional status enhances the efficacy of treatments and reduces their toxicity, as well as raising the patient's quality of life. Together with sarcopenia and tumour cachexia, anorexia and reduced caloric intake are the main causes of malnutrition in cancer patients. Anorexia-Cachexia Syndrome, or ACS, affects up to 80% of patients with advanced cancer (Inui 2002). ACS is a wasting syndrome leading to loss of skeletal muscle and fat, and an imbalance in the homeostatic system and thermoregulation. Patients affected with ACS consume energy reserves that hamper the body from generating heat through the breakdown of nutrients resulting in thermal stress.

Patients with advanced or terminal illness complain of feeling cold and are more sensitive to the perception of lower temperatures. The difficulty in maintaining body temperature is due to the impaired thermoregulatory capacity of the body arising from the additional calorie loss of ACS patients that aggravates their poor nutritional status. Thus, higher energy demands during colder months accelerates weight loss in advanced cancer patients. The buffering effect of warming has been observed in research with animal models of anorexia nervosa, where heightened AT prevented and reversed weight loss in animals deprived from food (Gutiérrez et al. 2008; Cerrato et al. 2012; Fraga et al. 2020; Roura et al. 2020; Fraga, Nogueiras, Fernø, Dieguez, Gutierrez, et al., 2021).

Similar to human cancer patients, tumour-bearing mice also appear to experience “feeling cold”. Thus, temperature preference studies, where mice were allowed to move between chambers maintained at different AT’s (22, 28, 30, 34, or 38 °C), have shown tumour-bearing mice preferred higher AT than non-tumour-bearing mice (Kokolusa Maegan, Capitanoa, Lee, Eng, Waight, et al., 2013). Moreover, housing mice injected with moderate doses of tumour cells at thermoneutral AT (30–31 °C), was associated to a remarkable reduction in tumour formation, growth rate, and metastasis (Kokolusa, et al., 2013).

The protective role of increased AT was reported in an ecological study of cancer death rates in the state of Florida from 2006 to 2010, with an average 3.4 fewer deaths per 100,000 people per one degree increment within the 19.8–24.8ºC temperature range of the study (Hart 2015).

The observation of an interaction between climate and human health dates back in time, with AT receiving the greatest attention among climatic factors. Accordingly, to two different reports of the Nongovernmental International Panel on Climate Change state: “*Medical science and observational research in Asia, Australia, Europe, and North America confirm that warming is associated with lower, not higher, temperature-related mortality rates*” [and that] *warmer temperatures lead to decreases in premature deaths due to cardiovascular and respiratory disease and stroke occurrences*” (Bezdek, Idso, Legates, & Singe, 2019, p.6). Furthermore, an analysis of monthly deaths of cardiovascular, respiratory, and digestive diseases found a 15% higher death rate in winter than in summer (Fernandez-Raga et al. 2010).

Likewise, seasonal variations have been reported in chronic heart failure (CHF) with greater CHF related morbidity and mortality during winter months in both northern (Boulay, Berthier, Sisteron, Gendreike & Gibelin, 1999; Feldman, Déry, Platt,, Déry, Kapetanakis, et al., 2004; Martinez-Selles. Garcia Robles, & Prieto, 2002; Stewart, McIntyre, Capewell, & McMurray, 2002), and southern hemispheres (Inglis et al. 2008). Moreover, a study of the seasonal impact on heart failure hospitalization outcomes in the U.S. showed hospitalizations, length of stay in hospital, and mortality were higher in winter than in summer, with age and sex being crucial moderators on these associations (i.e., inpatient mortality was only significant for patients aged ≥ 65 years, and females had a lower risk of inpatient mortality in summer (Akintoye, Briasoulis, Egbe, Adegbala, Alliu, et al., 2017).

The present study was contextualized within the research on the influence of natural seasonal weather conditions on oncological diseases. Cancer literature on climate is mainly focused on climate toxicity (Weadick, Keogh, Carroll, Boldrin, Mulroe, et al., 2023), and climate change associated heat waves, wildfires, droughts, flooding, and extreme weather, which increase "exposures to ultraviolet radiation, air pollution, disruptions in the food and water supply, environmental toxicants, and infectious agents" (Hiat & Beyeler, 2020, p. e519). To the best of our knowledge, there are no previous reports in the literature on seasonal influences on the evolution of bodyweight in patients with heterogeneous types of advanced cancer and ACS However, previous research has reported the buffering effect of increased ambient temperature on weight loss in an animal model of anorexia nervosa (Gutierrez et al., 2008; Fraga el al., 2020). Though the authors are aware that weight loss associated to oncological disease is an entity distinct from weight loss due to simple starvation, the main aim of the present study was to assess over a three-year period the seasonal influence of environmental temperature on the evolution of bodyweight in patients with advanced cancer and ACS, a wasting syndrome causing dramatic weight loss.

## Method

**Fig. 1:**
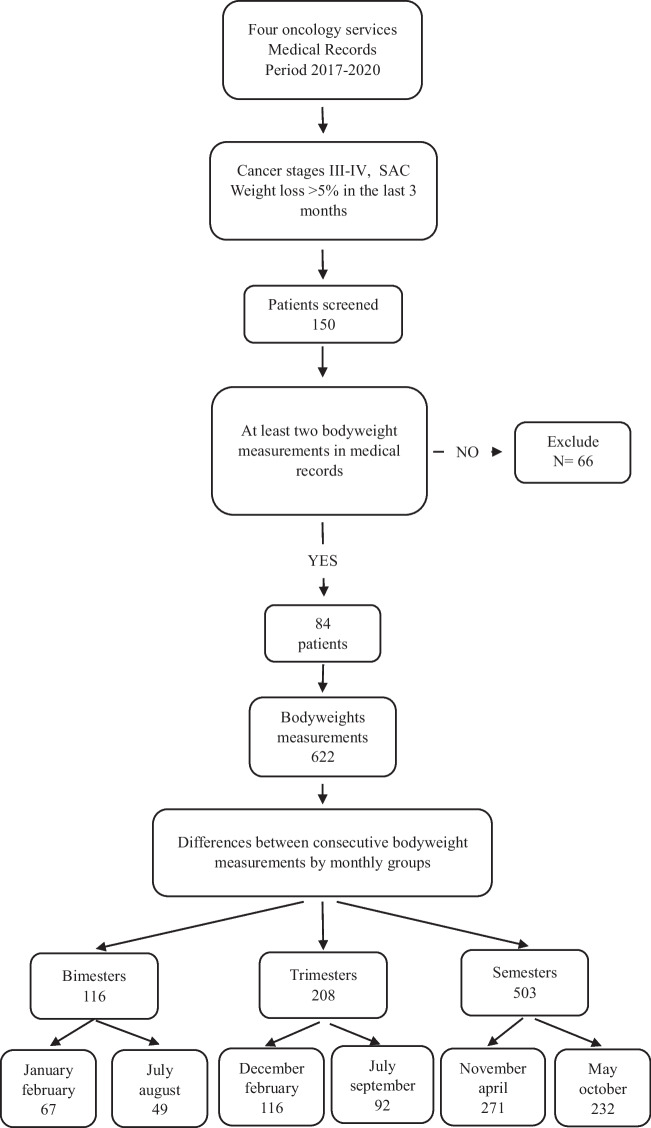
Flowchart showing patient inclusion criteria and the number of differences between consecutive body weight measurements included in the different monthly groups

As shown in the flowchart of Fig. 1, the initial sample consisted of 150 patients undergoing treatment in the oncology departments of four hospitals in Extremadura, Spain (Caceres, Badajoz, Mérida, and Llerena-Zafra) from 2017 to 2020. Inclusion criterion for this study were a diagnosis of oncological disease in stages 3 or 4 according to the AJCC Cancer Staging Manual (Amin et al. 2017), and ACS with weight loss according to the European Society of Clinical Nutrition and Metabolism (ESPEN) of more than 5% in the last 3 months. Cancer staging was determined by medical doctors from the oncological units of the four hospitals, and the data was gathered from the patients’ medical records.

The final sample was reduced to 84 patients after excluding patients whose clinical history contained less than two weight measures and/or an interval of more than 240 days between two consecutive weight measures. Data on sociodemographic characteristics (age, sex, place of residence) and clinical characteristics (disease, stage, main symptom, treatment, weight, date and place of death) were also obtained from notes contained in the clinical histories. All procedures were approved by Ethics Committee for Clinical Research of the Badajoz Health Area Management.

From the review of medical records, the first bodyweight record in 2017 was considered the initial weight, and the last bodyweight either due to death, or at the end of the study period in August 2020 was considered the final bodyweight. Body weight measurements were taken by medical staff at the four oncology services before lunch using the Asimed electronic scale (AMEDIC, S.L., Barcelona). Furthermore, the evolution of bodyweight throughout the 2017–2020 period was calculated as differences between consecutive weight measures which yielded a total of 622 differences (either weight gain, weight loss, or no change in bodyweight).

## Ambient temperature

The monthly average temperature of the four geographical areas involved in the study were obtained (Supplementary Table S1) from the official website of the Spanish State Meteorological Agency (AEMET, http://www,aemet,es/es/serviciosclimaticos/vigilancia_clima/resumenes?w=1&k=ext,). Thus, differences between consecutive weight measures were assigned to either a warm or cold season for different monthly groupings with significant differences in AT. Thus, as shown in Table 1, warm Bimesters (BIMs), included patients’ bodyweight measures taken during the months of July and August (mean AT of 26.6ºC), whereas cold BIMs (mean AT of 9.1ºC) included bodyweight measures obtained from December to January. Warm Trimesters (TRIMs) included measures recorded from July to September (mean AT of 25.7ºC), and cold TRIMs measures recorded from December to February (mean AT of 9.6ºC). Finally, warm Semesters (SEMs) extended from May to October (mean AT of 22.9ºC), and cold SEMs vs. from November to April (mean AT of 11.6ºC).

**Table 1:** Mean monthly temperature for Cold and Warm seasons during the period 2017–2020 for the geographic catchment areas in the Spanish Extremadura region involved in the study

	Cold	Warm	difference	t	*p*
Bimesters	9.08 ± 0.4	26.6 ± 0.5	17.5	31.7	< .0001
Trimesters	9.6 ± 1.3	25.7 ± 1.7	16.1	13.6	< .0001
Semesters	11.6 ± 2.2	22.9 ± 3.2	11.3	6.5	< .0001

For the analysis of the differences between warm and cold monthly groupings, bodyweight changes between any two consecutive measures were only considered if the maximum time gap between them had not elapsed. Thus, in the case of BIMs, a difference between two consecutive weight measures was valid if there was a time gap ≤ 80 days between them. In the case of TRIMs and SEMs, the respective time gaps were of ≤ 120 and ≤ 240 days.

### Statistical analysis

For the analysis of the relationship between environmental temperature and bodyweight a single factor ANOVA was performed. The *Z*-Score tests were used to compare differences in the percentages of patient weight gain or loss during warm and cold seasons. Comparisons for the statistical significance of relative frequencies were carried out using the chi-square statistic (Yates corrected, or Fisher’s exact test if necessary). Statistical analyses were performed using SPSS PSS statistical software (version 26.0; SPSS Inc., Chicago, IL).

## Results

As shown in Table 2, men doubled the number of women in the sample (age range: 37 to 91 years), with a higher percentage of patients over 65 years of age. When considering the two factors, sex and age, 56% of women in the sample were aged under 65 years, and 59.3% of men were aged over 65 years. However, this different distribution in terms of age and sex was not significant, χ^2^ (1, *N* = 84) = 1.66, *p* < 0.20.

**Table 2:** Sociodemographic characteristics of the sample studied (*N* = 84)

Variable	*N*	%	*p*
*Sex*			< .001
Male	59	70.2	
Female	25	29.8	
*Age*			n.s
< 65 years	38	45.2	
> 65 years	46	54.8	
Total	84	100	
*Sex/Age*			n.s
Male < 65 years	24	41	
Male > 65 years	35	59	
Female < 65 years	14	56	
Female > 65 years	11	44	
*Residence*			< .001
Urban	31	36.9	
Rural	53	63.1	
*Deaths during study*			< .001
No	28	33.3	
Yes	56	66.7	
*Place of death*			n.s
Home	23	41.1	
Hospital	33	58.9	

As for place of residence, nearly two thirds of the sample (63.1%) resided in rural areas, and the remainder (36.9%) resided in urban areas. At the final date of data collection, a significantly high percentage of patients, 66.7%, had died (*z* = 2.95, *p*. 001). However, with respect to casualties during the period of study there were no differences between both sexes, and regarding place of death.

The clinical characteristics of the sample are shown in Supplementary Table S2. A total of six different pathologies were present, with gastrointestinal tumours being the most frequent pathology (59%), followed by lung (12%), neck and brain, and genitourinary, whilst the lowest percentages were found in tumours of the breast and the female reproductive system. Additionally, according to the classification of the National Cancer Institute (NCI), a significantly higher percentage of patients (67.9%) suffered from digestive oncological diseases (tumours affecting the digestive tract) and those of the head, neck, and brain, in comparison to other oncological diseases (*z* = 3.16, *p* < 0.001).

There was a considerable variance in bodyweight measures in the hospital records. As shown in Table 3, there was a wide range of bodyweight measures per patient (from 2 to 27). However, there were no differences in the number of measures for those dying during the study in comparison to survivors, *F (*1, 83) = 0.796, *p* = 0.38, nor with respect to changes in bodyweight during the period of study *F (*1, 83) = 0.391, *p* = . 534.

**Table 3:** Bodyweight measures in patient medical records in four hospitals of Extremadura region during the period 2017-2020.

	Whole Sample (*N* = 84)	Males (*N* = 59)	Females (*N* = 25)	Deceased (*N* = 56)	Survivors (*N* = 28)
Initial weight	69.3 ± 15.3	72.3 ± 13.6	68.1 ± 13.4	68.1 ± 15.3	71.7 ± 15.3
Final weight	66.4 ± 15.6	62.2 ± 6.9	62.3 ± 3.4	64.5 ± 14.6	70.2 ± 17.2
Weigth records range	2–27	2–27	2–25	2–19	2–27
Average records	16.7	8.05	9.19	8.14	8.93

Table 4 shows the number of observations made in cold and warm monthly grouping categories (BIMs, TRIMs, and SEMs), and Table 5 shows the weight gain and loss percentages observed in the differences between consecutive weights during the cold and warm seasons. Although the number of observations in the cold seasons exceed that for the warm seasons (see Table 5), this difference was only significant for the semester (*z* = 1.70, *p* = 0.04).

**Table 4:** Number of differences between consecutive bodyweight measures in medical records for each monthly grouping and patient characteristics in four hospitals of Extremadura region during the period 2017–2020

	Bimester (*N* = 116)	Trimester (*N* = 208)	Semester (*N* = 503)
Cold months	67 (57.8%)	116 (55.8%)	271 (53.9%)
Warm months	49 (42.2%)	92 (44.2%)	232 (46.1%)
Sex			
Male	81 (69.8%)	142 (68.3%)	354 (70.4%)
Female	35 (30.2%)	66 (31.7%)	149 (29.6%)
Age			
≤ 65	64 (55.2%)	79 (38%)	263 (52.3%)
> 65	52 (44.8%)	129 (62%)	240 (47.7%)
Exitus			
No	46 (39.7%)	31 (24%)	192 (38.2%)
Yes	70 (60.3%)	98 (76%)	311 (61.8%)
Deaths			
Home	14 (20%)	115 (55.3)	86 (27.7%)
Hospital	56 (80%)	93 (44.7)	225 (72.3%)
Location			
Urban	40 (34.5%)	74 (35.6%)	178 (35.4%)
Rural	76 (65.5%)	134 (64.4)	325 (64.4%)

**Table 5:** Number and percentages of gain and weight losses between two consecutive measurements for different monthly groupings during the period 2017–2020 for the patients included in the study

	Cold Bimester (67)	Warm Bimester (49)	Z	*p*
Weight loss	33(49.25%)	15(30.61%)	2.014	0.044
Weight gain	26 (38.81%)	26(53.06%)	-1.525	0.127
No change	8(11.94%)	8(16.33%)	-0.677	0.498
	Cold Trimester (116)	Warm Trimester (92)		
Weight loss	60(51.72%)	38 (41.30%)	1.495	0.134
Weight gain	42(36.21%)	40(43.48%)	-1.066	0.286
No change	14(12.07%)	14(15.22%)	-0.661	0.508
	Cold Semester (270)	Warm Semester (232)		
Weight loss	134(49.63%)	98(42.28%)	1.655	0.097
Weight gain	96(35.56%)	96(41.38%)	-1.339	0.180
No change	40(14.81%)	38(16.38%)	-0.482	0.629

Regarding bodyweight evolution there was a significant difference in weight loss across warm and cold BIMs. The differences in the weight loss percentages between consecutive weight measures was higher during the cold season from December to January (mean temperature of 9ºC) in comparison to the warmest months of July and August (mean temperature of 27ºC), *z* = 2.01, *p* = 0.04. Although the same trend of greater weight loss percentages was observed for both the cold TRIMs (December to February) and SEMs (November to April) in comparison to warm TRIMs (June to August) and SEMs (May to October), these differences did not reach statistical significance (*z* = 1.50, *p* = 0.13, and *z* = 1.66, *p* = 0.10, respectively).

However, the higher weight loss percentages were not reflected in differences in the average changes between consecutive weight measures for warm and cold periods. Thus, although patients gained an average of 389 g in the months of July and August (see Supplementary Table S3), as compared to the average loss of -242 gr in the cold months (December and January), the net difference of 631 g did not reach the significance level of 0.05, *F (*1, 115) = 3.416, *p* = 0.07. Neither differences between the cold and warm TRIMs were significant, *F (*1, 206) = 1.163) = *p* = 0.28, nor in the case of the SEMs, *F (*1, 501) = 1.92 *p* = 0.17.

The negative impact of cold periods was more marked in men than in women. Thus, the weight loss percentage for men during the cold seasons was higher than for warm seasons (52% vs. 30%, *z* = 1.94 p = 0.05; 61% vs. 34, %, *z* = 2.14, *p* = 0.03; and 50% vs. 42%, *z* = 1.65, *p* = 0.10, percentages for cold vs warm BIMs, TRIMs, and SEMs, respectively). In contrast, the opposite trend was found in women, that is, the weight gain percentage was higher during warmer months (39% vs. 53%, z = 1.52, *p* = 0.13; 34% vs. 61%, *z* = 2.137, *p* = 0.03, and 35 0.6% vs. 41.4%, *z* = 1.34, *p* = 0.13, percentages for cold vs warm BIMs, TRIMs, and SEMs, respectively). However, these differences were not reflected in differences in mean bodyweight except in the comparison of SEMs where the tendency to gain weight in women in the warmer months was significantly greater than in men, (54.7% vs. 36.3%, *z* = 2.540, *p* = 0.01), with the difference nearly approaching statistical significance, F (1, 501) = 3,530 *p* = 0.06.

As shown in Table 1, 54 patients died during the study period. However, there were neither statistically significant differences between the number of deaths across warm (30) and cold (24) seasons (*z* = 0.68, *p* = 0.50), nor differences across BIMs, TRMIs, and SEMs, χ2 (2, *N* = 54) = 0.63, *p* = 0.73.

In contrast, temperature had a sizeable impact on the weight trend of patients who died during the study. In general, in all BIMs, TRIMs and SEMs, higher weight loss percentages were observed during cold seasons in comparison to warm seasons (52% vs. 31%, *z* = 1.75, *p* = 0.08; 54% vs. 41%, *z* = 1.41, *p* = 0.16; 52% vs. 41%, *z* = 1.92, *p* = 0.06, for BIMS, TRIMS, and SEMS, respectively), and a significant weight gain tendency during warm seasons (58% vs. 34%, *z* = 1.92, *p* = 0.05; 49% vs. 32%, *z* = 1.93, *p* = 0.05; and 46% vs. 33%, *z* = 2.23, *p* = 0.02, for BIMS, TRIMS, and SEMs). Nevertheless, only differences for SEMs were confirmed by the corresponding ANOVAS for bodyweight *F (*1, 309) = 6.330 *p* = 0.01, but failed to reach statistical significance for BIMs, *F (*1.69) = 2,246, *p* = 0.14, or TRIMs, *F (*1, 127) = 3,062, *p* = 0.08. Moreover, a significant interaction was found for SEMs between temperature (cold/warm), and mean weight *F (*1, 499) = 6.06, *p* = 0.01, which reflected a higher weight loss percentage in the cold semester indicated above, whilst in the warm semester, on the contrary, the weight gain was higher than in the cold semester, *z* = 2.23, *p* = 0.02. Furthermore, an ANOVA performed for deceased patients revealed that the difference in weight loss at home in the cold versus warm SEMs did not reach statistical significance, *F (*1.86) = 0.826, *p* = 0.365. However, in hospital deaths the difference was significant *F (*1, 223) = 5,521, *p* = 0.02.

Finally, the effect of temperature was more pronounced in patients living in rural versus urban settings. Although patients in both urban and rural locations lost weight during the cold months, weight gain prevailed in hot months. However, this trend did not reach statistical significance neither for the BIMs, nor TRIMs [*F (*1, 39) = 0.474 *p* = 0.50, and *F (*1, 75) = 3.12, *p* = 0.08; and *F (*1, 72) = 0.13 *p* = . 72, and *F (*1, 132) = 1,40 *p* = 0.24, for urban and rural residence, respectively]. In comparison, in the ANOVA performed for the group of patients living in rural areas, the ANOVA performed for cold and hot SEMs was statistically significant, *F (*1,323) = 3,924, *p* = 0.05.

## Discussion

**Fig. 2:**
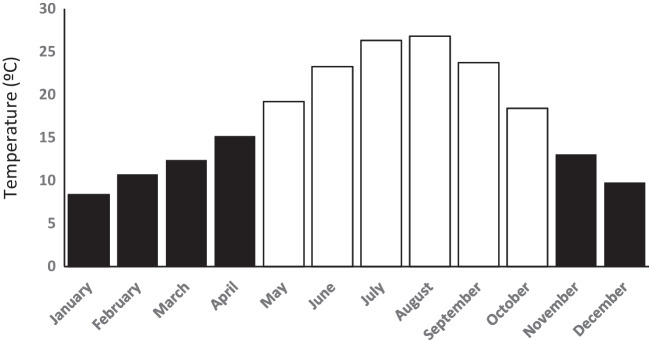
Mean monthly temperature during the period 2017–2020 for the hospital geographical catchment areas where patients were recruited from the region of Extremadura, southwestern Spain. Cold months in black and warm months in white

To determine the influence of AT on the evolution of bodyweight in patients with advanced oncological disease and ACS in view of the data shown in Fig. 2, monthly groupings of BIMs, TRIMs and SEMs were used to glean meaningful differences in environmental temperature. This classification was preferred to the conventional yearly seasonal classification of spring, summer, autumn, and winter defined by astronomical equinoxes and solstices, or meteorologically, where seasons begin earlier and correspond to full three-month periods, given that conventional seasons misclassify April and October. Although conventional astronomical and meteorological seasonal classifications are appropriate with respect to sunlight, this classification is incorrect if the parameter of interest is AT, since September and October, despite having lower average daylight hours, are warmer months than March and April, as shown in Supplementary Table S1. This is due to a phenomenon known as the "seasonal lag" of temperature relative to insolation. It takes time for change in the previous season's average temperatures of land and water surfaces, so summer heat does not fully fade in the case of October, whereas the opposite occurs in April with the coldness of winter.

The analyses partially supported a pattern of a negative influence of cold seasons, which was more marked in men, for patients deceased during the study, and in particular for urban residents who had died in hospital. The comparison of weight loss and gain percentages across cold and warm BIMS, where temperature differences between cold and warm seasons were high (17.5º C), showed a clear effect of AT for the whole sample (*p* = 0.04), with higher weight loss percentages in men (*p* = 0.05), and for patient deceased during the study (*p* = 0.04). However, this pattern was not supported in the comparison of the average weight loss and gain differences between consecutive weight measures across cold and warm seasons, where the corresponding ANOVAs failed to reach the alpha 0.05 level of statistical significance (*p* = 07, 13, and 0.08 for the whole sample, men, and deceased, respectively).

Sex differences were also clear for TRIMs and SEMs. In the case of TRIMs, although weight loss and gain percentages across cold and warm season were not significantly different in terms of net weight differences, the ANOVA yielded a significant difference in weight gains for women during warm seasons (*p* = 03). As for SEMs, an interaction was observed for deceased patients who tended to lose weight during the cold season while gaining weight during the warm season. In terms of the relationship between age and weight loss, as shown in Table S3, patients over 65 years of age showed less weight loss during warm periods, being most notable in warm quarters and semesters, although these differences did not reach significance (0.17, 0.07 and 0.07 for BIM, TRI, and SEM, respectively).

As shown in Table S3, negative values were the norm in the cold seasons’ column for BIMs, TRIMs, and SEMs, indicating a decrease in bodyweight except for survivors. This pattern was more marked in the BIMs, where in the warm season the evolution of bodyweight was the opposite to that in the cold season. In contrast, in the BIM warm seasons’ column all values were positive, which meant weight gain. In the comparison of the warm and cold BIM columns, the smallest difference (-451 g) was found in the urban locality variable, and the largest difference was observed in patients who died at home during the study, which amounted to 1.211 g.

As for the limitations of the study, it would have been useful to have had monthly bodyweight measurements, but the inconsistent regularity in patient weight measurements prevented a monthly analysis of the relationship between AT and weight loss. As can be seen in Table 4 for the three-year period of the study, the average number of bodyweight measurements was roughly 17, less than half of the 36 measurements had the weighing process been undertaken on a monthly basis, a circumstance that did not occur in any patient, with the highest number of recorded measurements in surviving patients being 27, as shown in Table 4. Another limitation of this study was the lack of information on diets as a moderating factor for weight loss/gain. In the patients’ medical records, nutritional drink supplements were always prescribed since ACS in cancer patients cannot be reversed by conventional nutritional support (Fearon et al. 2011), and evidence from experimental animal models (van de Worp, Schols, Theys, van Helvoort, & Langen, 2020) has recommended nutritional intervention as an integral part of multimodal therapy (Arends, Baracos, Bertz, Bozzetti, Calder, et al., 2017; Prado et al. 2020). A further limitation of this study was the lack of the patients’ weight measurements closest to the date of diagnosis prior to admittance to the study. This lack of data hindered comparisons with the average weight of the male/female population of the region. Nevertheless, the weight status of the patients had been determined at the start of the study in order to satisfy the inclusion criteria of a weight loss of more than 5 kg in the last three months (see Fig. 1). As shown in Table S2, patients continued losing weight during the study period. However, the feeding difficulties associated to these pathologies did not show significant differences in weight loss, *t* (82) = 1.0553, *p* = 0.2944. Finally, another limitation of the study was the absence of specific information on the treatment administered to each patient though all patients were actively receiving chemotherapy, radiation therapy, or both.

It should be noted that experimental studies have shown that a heated environment of 32ºC reversed hypothalamic MC4 overexpression in an animal model of anorexia nervosa (Gutiérrez et al. 2009). Given the pivotal role of the melanocortin-4 receptor (MC4) in the control of appetite (Adan, Tiesjema, Hillebrand, la Fleur, Kas MJ, et al., 2006), and cachexia (Foster & Chen 2007), and considering that mean AT in the catchment area where patients were recruited exceeded 26ºC during warmer months (July and August), it is reasonable to believe that the melanocortin system may be involved in the modulating effect of AT on bodyweight observed in our sample of patients with advanced oncological disease and ACS.

## Conclusion

Weight loss predicts a poor prognosis for cancer patients and most patients with advanced cancer do not appear to benefit from nutritional supplementation (Jatoi & Loprinzi, 2001). Our data show that weight loss is not entirely driven by the disease process but seems to be also modulated by ambient temperature. Preclinical research has already shown that ambient temperature was associated to a remarkable reduction in tumor formation, growth rate, and metastasis (Kokolusa, et al., 2013). Though indoor thermal comfort is provided through heating, ventilation, and air-conditioning systems, outdoor AT has been extensively used in epidemiological research as a surrogate for personal exposure to heat and cold (Basu, 2002). In this study, AT was found to be a key factor influencing body weight changes in patients with advanced oncological disease and ACS. However, the two main limitations of the study were the lack of information on diets as a moderating factor on weight loss/gain, and the lack of measurements on the patients’ weight closest to the date of diagnosis before admittance to the study. In terms of the practical implications, it remains an unresolved question whether a heightened ambient temperature could be employed as a further strategy for strengthening the survival of patients with advanced cancer. and ACS.

## Supplementary Information

Below is the link to the electronic supplementary material.

Supplementary file1 (DOCX 16 KB)

Supplementary file2 (DOCX 14 KB)

Supplementary file3 (DOCX 23 KB)

## Data Availability

The datasets generated during and/or analysed during the current study are available from the corresponding author on reasonable request.
