# Supplementary material for: Ambient temperature modulates body weight changes in patients with advanced oncological diseases and anorexia cachexia syndrome
Source: Int J Biometeorol. 2023 Jul 4;67(9):1451–9. doi: 10.1007/s00484-023-02513-4 (PMC10432328; doi:10.1007/s00484-023-02513-4)
Supplement: Supplementary file 1 — Supplementary file1 (DOCX 16 KB) [file 484_2023_2513_MOESM1_ESM.docx]

Supplementary Table S1. Mean monthly temperature for individual years and the period 2017-2020 in the hospital catchment areas in Extremadura region involved in the study.

|  | 2017 | | | 2018 | | 2019 | | 2020 | 2017-2020 | |  |
| --- | --- | --- | --- | --- | --- | --- | --- | --- | --- | --- | --- |
| January | | 7.7 | 8.6 | | 8.1 | | 9 | | | 8.4 | |
| February | | 11.2 | 8.7 | | 10.6 | | 12.4 | | | 10.7 | |
| March | | 12.7 | 10.9 | | 13.2 | | 12.7 | | | 12.4 | |
| April | | 17.2 | 14.3 | | 14.1 | | 15 | | | 15.2 | |
| May | | 20.4 | 17.3 | | 20.5 | | 18.5 | | | 19.2 | |
| June | | 26.2 | 22.3 | | 22.1 | | 22.4 | | | 23.3 | |
| July | | 26.5 | 24.1 | | 25.7 | | 28.9 | | | 26.3 | |
| August | | 26.6 | 27.9 | | 26.2 | | 26.5 | | | 26.8 | |
| September | | 22.8 | 25.5 | | 23.3 | | 23.2 | | | 23.7 | |
| October | | 21 | 17.8 | | 18.3 | | 16.5 | | | 18.4 | |
| November | | 13 | 12.4 | | 12.6 | | 14.2 | | | 13.1 | |
| December | | 9 | 9.4 | | 11.2 | | 9.5 | | | 9.8 | |
|  | |  |  | |  | |  | | |  | |
