# Supplementary material for: Ambient temperature modulates body weight changes in patients with advanced oncological diseases and anorexia cachexia syndrome
Source: Int J Biometeorol. 2023 Jul 4;67(9):1451–9. doi: 10.1007/s00484-023-02513-4 (PMC10432328; doi:10.1007/s00484-023-02513-4)
Supplement: Supplementary file 2 — Supplementary file2 (DOCX 14 KB) [file 484_2023_2513_MOESM2_ESM.docx]

Supplementary Table S2. Type of cancer and feeding difficulties characteristics in the sample (N = 84)

| **Variable** | **N** | **%** | **Weight loss** |
| --- | --- | --- | --- |
| Cancer Pathology |  |  |  |
| Gastrointestinal | 49 | 59.0 | -2.9 ± 9.2 |
| Lung | 10 | 12.0 | 1.8 ± 4.8 |
| Genitourinary | 8 | 9.6 | -7.7 ± 9.8 |
| Brain, neck | 8 | 9.6 | -5.8 ± 6.8 |
| Breast | 4 | 4.8 | 2.0 ± 9.2 |
| Gynaecologic | 4 | 4.8 | -4.7 ± 12.9 |
| Feeding difficulties |  |  |  |
| Present | 57 | 67.9 | -3.6 ± 9.5 |
| Absent | 27 | 32.1 | -1.3 ± 8.6 |
|  |  |  |  |
