# Supplementary material for: Ambient temperature modulates body weight changes in patients with advanced oncological diseases and anorexia cachexia syndrome
Source: Int J Biometeorol. 2023 Jul 4;67(9):1451–9. doi: 10.1007/s00484-023-02513-4 (PMC10432328; doi:10.1007/s00484-023-02513-4)
Supplement: Supplementary file 3 — Supplementary file3 (DOCX 23 KB) [file 484_2023_2513_MOESM3_ESM.docx]

Supplementary Table S3. Differences between consecutive patient’s bodyweight records for cold and warm seasons in the different monthly groups for the period 2017-2020.

|  | COLD | | WARM | | | |  |  |
| --- | --- | --- | --- | --- | --- | --- | --- | --- |
|  | **N*** | **Mean ± sd^#^** | | **N*** | **Mean ± sd** | **F** | | **p** |

| Bimesters | Total sample | | 67 | -0.242 ± 1.95 | | 49 | | 0.389 ± 1.81 | | 3.134 | | .089 | |  |
| --- | --- | --- | --- | --- | --- | --- | --- | --- | --- | --- | --- | --- | --- | --- |
|  | Male | | 48 | -0.262 ± 2.09 | | 33 | | 0.197 ± 1.70 | | 1.089 | | .300 | |  |
|  | Female | | 19 | -0.189 ± 1.50 | | 16 | | 0.787 ± 2.03 | | 2.551 | | .120 | |  |
|  | ≤ 65 | | 33 | -0.030 ± 1.99 | | 31 | | 0.438 ± 1.83 | | 0.953 | | .333 | |  |
|  | >65 | | 34 | -0.447 ± 1.92 | | 18 | | 0.305 ± 1.83 | | 1.864 | | .178 | |  |
|  | Survivors | | 23 | 0.082 ± 1.97 | | 23 | | 0.513 ± 1.94 | | 0.554 | | .461 | |  |
|  | Deceased | | 44 | -0.411 ± 1.94 | | 26 | | 0.280 ± 1.72 | | 2.246 | | .139 | |  |
|  | Death at home | | 11 | -0.045 ± 2.29 | | 3 | | 1.160 ± 1.75 | | 0.707 | | .417 | |  |
|  | Death at Hospital | | 33 | -0.533 ± 1.83 | | 23 | | 0.165 ± 1.72 | | 2.057 | | .157 | |  |
|  | Urban | | 22 | -0.390 ± 1.94 | | 18 | | 0.061 ± 2.21 | | 0.474 | | .495 | |  |
|  | Rural | | 45 | -0.168 ± 1.97 | | 31 | | 0.580 ± 1.55 | | 3.120 | | .081 | |  |
| Trimesters | Total sample | 116 | | | -0.384 ± 1.94 | 92 | -0.081 ± 2.09 | | | | 3.134 | | .282 | |
|  | Male | 78 | | | -0.342 ± 2.01 | 64 | -0.275 ± 2.07 | | | | 1.089 | | .845 | |
|  | Female | 38 | | | -0.471 ± 1.80 | 28 | 0.360 ± 2.10 | | | | 2.551 | | . .090 | |
|  | ≤ 65 | 54 | | | -0.246 ± 2.12 | 61 | -0.249 ± 2.15 | | | | 0.953 | | . .994 | |
|  | >65 | 62 | | | -0.504 ± 1.78 | 31 | 0.248 ± 1.96 | | | | 1.864 | | .067 | |
|  | Survivors | 38 | | | 0.131 ± 1.83 | 41 | -0.175 ± 2.15 | | 0.554 | | | | .499 | |
|  | Deceased | 78 | | | -0.635 ± 1.90 | 51 | -0.050 ± 2.00 | | 2.246 | | | | .083 | |
|  | Death at home | 21 | | | -0.738 ± 2.06 | 10 | -0.210 ± 2.44 | | 0.707 | | | | .535 | |
|  | Death at hospital | 57 | | | -0.598 ± 1.93 | 41 | 0.439 ± 1.99 | | 2.057 | | | | .112 | |
|  | Urban | 38 | | | -0.523 ± 1.93 | 36 | -0.350 ± 2.25 | | 0.126 | | | | .723 | |
|  | Rural | 78 | | | -0.316 ± 1.95 | 56 | -0.091 ± 1.98 | | 1.399 | | | | .239 | |

|  |  |  |  |  |
| --- | --- | --- | --- | --- |

| Semesters | Total sample | 271 | -0.444 ± 2.17 | 232 | -0.181 ± 2.03 | 1.920 | .166 |
| --- | --- | --- | --- | --- | --- | --- | --- |
|  | Male | 186 | -0.579 ± 2.12 | 168 | -0.278 ± 1.97 | 1.908 | .168 |
|  | Female | 85 | -0.148 ± 2.27 | 64 | 0.070 ± 2.19 | 0.347 | .557 |
|  | ≤ 65 | 130 | -0.301 ± 2.33 | 133 | -0.250 ± 2.00 | 0.036 | .849 |
|  | >65 | 141 | -0.575 ± 2.01 | 99 | -0.089 ± 2.07 | 3.294 | .071 |
|  | Survivors | 91 | -0.044 ± 1.99 | 101 | -0.381 ± 2.08 | 1.306 | .255 |
|  | Deceased | 180 | -0.646 ± 2.23 | 131 | -0.028 ±1.99 | 6.334 | .012 |
|  | Death at home | 53 | -0.926 ± 2.63 | 33 | -0.442 ± 1.94 | 0.829 | .365 |
|  | Death at hospital | 127 | -0.529 ± 2.05 | 98 | 0.111 ± 2.00 | 5.521 | .020 |
|  | Urban | 85 | -0.371± 2.30 | 93 | -0.407 ± 2.23 | 0.011 | .917 |
|  | Rural | 186 | -0.477± 2.09 | 139 | -0.030 ± 1.88 | 3.928 | .048 |

**^#^** Number of differences computed between consecutive weight measurements by season.

* kilograms
